# Adults with well‐healed burn injuries have lower pulmonary function values decades after injury

**DOI:** 10.14814/phy2.15264

**Published:** 2022-05-17

**Authors:** Joseph C. Watso, Steven A. Romero, Gilbert Moralez, Mu Huang, Matthew N. Cramer, Manall F. Jaffery, Bryce N. Balmain, Daniel P. Wilhite, Tony G. Babb, Craig G. Crandall

**Affiliations:** ^1^ Department of Internal Medicine University of Texas Southwestern Medical Center Dallas Texas USA; ^2^ Institute for Exercise and Environmental Medicine Texas Health Presbyterian Hospital Dallas Dallas Texas USA; ^3^ Department of Applied Clinical Research School of Health Professions University of Texas Southwestern Medical Center Dallas Texas USA; ^4^ Department of Physiology & Anatomy University of North Texas Health Science Center Fort Worth Texas USA

**Keywords:** body surface area, inhalation injury, lung diffusing capacity, lung volumes, spirometry

## Abstract

Sub‐acute (e.g., inhalation injury) and/or acute insults sustained during a severe burn injury impairs pulmonary function. However, previous work has not fully characterized pulmonary function in adults with well‐healed burn injuries decades after an injury. Therefore, we tested the hypothesis that adults with well‐healed burn injuries have lower pulmonary function years after recovery. Our cohort of adults with well‐healed burn‐injuries (*n* = 41) had a lower forced expiratory volume in one second (Burn: 93 ± 16 vs. Control: 103 ± 10%predicted, mean ± SD; *d* = 0.60, *p* = 0.04), lower maximal voluntary ventilation (Burn: 84 [71–97] vs. Control: 105 [94–122] %predicted, median [IQR]; *d* = 0.84, *p* < 0.01), and a higher specific airway resistance (Burn: 235 ± 80 vs. Control: 179 ± 40%predicted, mean ± SD; *d* = 0.66, *p* = 0.02) than non‐burned control participants (*n* = 12). No variables were meaningfully influenced by having a previous inhalation injury (*d* ≤ 0.44, *p* ≥ 0.19; 13 of 41 had an inhalation injury), the size of the body surface area burned (*R* ^2^ ≤ 0.06, *p* ≥ 0.15; range of 15%–88% body surface area burned), or the time since the burn injury (*R* ^2^ ≤ 0.04, *p* ≥ 0.22; range of 2–50 years post‐injury). These data suggest that adults with well‐healed burn injuries have lower pulmonary function decades after injury. Therefore, future research should examine rehabilitation strategies that could improve pulmonary function among adults with well‐healed burn injuries.

## Graphical abstract

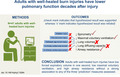

## 1 INTRODUCTION

Advances in the sub‐acute and acute care of burn injuries have improved rates of survival (Porter et al., 2015; Wolf et al., 2018). However, survivors of severe burn injuries are at an increased risk of long‐term complications (Peck, 2011; Duke et al., 2015, 2016, 2017; Jeschke et al., 2008, 2011; Stanojcic et al., 2018). Poor respiratory function, as observed in children, following discharge from intensive care (Desai et al., 1993) may contribute to epidemiological findings of low cardiorespiratory fitness and physical activity levels in survivors of severe burn injuries later in life (Dodd et al., 2017; Duke et al., 2017; Ferrando et al., 1997; Ganio et al., 2015; Willis et al., 2011). Together, these findings create a strong need to fully understand the physiological consequences of severe burn injuries; specifically, there are several unknowns related to the long‐term effects of severe burn injuries on pulmonary function in adults.

Various lines of evidence suggest that adults who have sustained severe burn injuries have reduced spirometry values and lung volumes. For example, after a burn injury, adults have lower values for forced expiratory volume in one second (FEV_1_), forced vital capacity (FVC), functional residual capacity (FRC), forced expiratory flow at 25%–75% (FEF_25–75%_), and total lung capacity (TLC) (Grisbrook et al., 2012; Nylen et al., 1995; Özkal et al., 2021; Whitener et al., 1980; Won et al., 2021). Additionally, previous studies have conflicting findings regarding whether FEV_1_/FVC and/or maximal voluntary ventilation (MVV) is reduced in adults with well‐healed burn injuries (Grisbrook et al., 2012; Özkal et al., 2021; Willis et al., 2011; Won et al., 2021). Studies have also reported that lung diffusing capacity is reduced in adults with well‐healed burn injuries when compared with non‐burn injured individuals (Desai et al., 1993; Grisbrook et al., 2012; Mlcak et al., 1995, 1998; Nylen et al., 1995; Suman et al., 2002; Won et al., 2021). However, lung diffusing capacity adjusted for alveolar volume has not been reported for this population, which limits our ability to interpret these data (Cerfolio & Bryant, 2009). Further, it is unclear whether a prior burn injury would affect other important measures of pulmonary function, such as airway resistance (Kaminsky, 2012). Lastly, much of the data detailed above were collected within the three years after an injury (Desai et al., 1993; Mlcak et al., 1995; Özkal et al., 2021; Suman et al., 2002; Whitener et al., 1980; Won et al., 2021) and the three longer follow‐up studies (data collected decades after injury) to date are limited by modest sample sizes (e.g., 7–9 patients per study) (Grisbrook et al., 2012; Nylen et al., 1995; Willis et al., 2011).

Observed detriments in pulmonary function among adults with well‐healed burn injuries could be due to a combination of factors. Such reasons include direct effects of inhalation injuries, respiratory injury via burns to the thoracic region, structural changes in the mechanical properties of the bronchial tree and lungs, hypermetabolism, hyperinflammation, transient reductions in surfactant, respiratory muscle weakness, noncardiogenic pulmonary edema, and/or prolonged bed rest during the critical recovery period of the burn injury (Bourbeau et al., 1996; Desai et al., 1993; Hart et al., 2000; Jeschke et al., 2011; Mlcak et al., 1998; Pavoni et al., 2010; Saltin et al., 1968; Stanojcic et al., 2018; Stephenson et al., 1975; Whitener et al., 1980; Won et al., 2021). While these mechanisms have been closely studied in the days, weeks, and months following a burn injury, it is unclear whether the observed detriments in pulmonary function are present years (or decades) after a burn injury.

Gaining a better understanding of the pathophysiology in the respiratory system among adults with well‐healed burn injuries will aid in developing specific rehabilitation strategies. However, it is unclear what facets of pulmonary dysfunction are present among adults with well‐healed burn injuries because a comprehensive pulmonary function testing battery has not been completed in a large cohort of this patient population. Therefore, to address this knowledge gap, we tested the hypothesis that adults with well‐healed severe burn injuries would score below normative ranges when assessed using a comprehensive pulmonary function testing battery. We also performed pulmonary function testing in non‐burn injured adults to serve as an additional comparator to burn‐injury survivors. Lastly, in addition to characterizing pulmonary function among a large group of adults with well‐healed burn injuries, we sought to explore whether the history of an inhalation injury, the size of body surface area burned, or the time since burn injury was associated with the expected impairments in pulmonary function, given their potential influence on pulmonary function (Bourbeau et al., 1996).

## 2 MATERIAL AND METHODS

The pulmonary function measures presented herein are unique, yet complementary, to prior published data from this cohort of burn‐injury survivors that addressed hypotheses related to aerobic capacity and physical function (Huang et al., 2020; Romero et al., 2019).

### 2.1 Ethical approval

This study protocol and informed consent were approved by the Institutional Review Boards of the University of Texas Southwestern Medical Center and Texas Health Presbyterian Hospital Dallas (Approval identifier: STU‐042014–060). All procedures conformed to standards outlined in the Declaration of Helsinki. Participants were fully informed, both verbally and in writing, of all the experimental procedures and the potential risks of participation before providing informed written consent.

### 2.2 Participants

We tested 41 individuals with well‐healed burn injuries and 12 non‐burned control participants between 2014 and 2019. The control participants were recruited from the Dallas, TX area using flyers and advertisements, while the burn survivors were recruited throughout North America. All adults with well‐healed burn injuries were studied at least 2 years post‐injury and none had musculoskeletal impairments that limited physical activity. Based on responses to a questionnaire, both adults with well‐healed burn injuries and control adults were sedentary and had not participated in a consistent/structured exercise training regimen at any time over the prior 12 months. Information related to burn injuries (e.g., presence of inhalation injury) was self‐reported in a medical history questionnaire. All participants were between 18 and 60 years old. Exclusion criteria for all participants included current smoker, pregnancy, breastfeeding, or any overt immune, pulmonary, renal, hepatic, cardiovascular, or gastrointestinal disease. Individuals with controlled hypertension or controlled hypercholesterolemia (medication usage detailed in the RESULTS section below) were permitted to enroll.

### 2.3 Pulmonary function assessments

We asked participants to abstain from caffeine, nutritional supplements, over‐the‐counter medications, alcohol, and physical activity for 24 h before testing. We allowed participants to take prescription medications, if necessary, before testing. All participants completed standard spirometry, MVV, lung volume, diffusing capacity, and airway resistance assessments (model 6200 body plethysmograph: SensorMedics, Yorba Linda, CA) according to American Thoracic Society guidelines (Graham et al., 2017, 2019) in a temperature‐controlled laboratory (~22°C) at the Institute for Exercise and Environmental Medicine in Dallas, TX. Percent predicted values were determined from the following published prediction equations based on normative values: (Hankinson et al., 1999) For spirometry data; (Kory et al., 1961) for MVV in male adults; (Grimby & Soderholm, 1963) for MVV in female adults; (Goldman & Becklake, 1959) for lung volumes; (Stanojevic et al., 2017) for transfer factor of the lung for carbon monoxide (TL_CO_); (Burrows et al., 1961) for lung diffusing capacity divided by alveolar volume (DL_CO_/V_alv_); and (Dubois et al., 1956) for airway resistance. Additionally, we used the National Health and Nutrition Examination Survey (NHANES) III age‐, sex‐ and ethnicity‐specific reference values (Hollowell et al., 2005) for hemoglobin (Hb) concentrations to calculate hemoglobin‐adjusted TL_CO_ using: TL_CO_[predicted for Hb] = TL_CO_[predicted] × (1.7Hb_measured_ / (0.7Hb_reference_ + Hb_measured_)) (Graham et al., 2017).

### 2.4 Data and statistical analysis

We did not conduct an *a priori* power analysis for the objectives addressed in the manuscript. We compared screening and pulmonary function data between the non‐burned control group and adults with well‐healed burn injuries using unpaired, two‐tailed *t*‐tests or Mann‐Whitney tests when data failed (*p* < 0.05) the Shapiro‐Wilk normality test. For all such non‐parametric analyses, we report median [interquartile range] instead of mean ± SD. We compared the percent predicted values within each group using one‐sample *t*‐tests (theoretical mean of 100) or Wilcoxon signed‐rank tests (theoretical median of 100) when data failed (*p* < 0.05) the Shapiro–Wilk normality test. Additionally, we compared the proportion of adults with values below the lower limit of normal (i.e., 80%) between groups using Fisher's exact tests for certain key variables. We analyzed these data using GraphPad Prism 9.3 (GraphPad Software Inc., La Jolla, CA, USA). We did not create a dichotomous line of significance/non‐significance (Curran‐Everett, 2020; Gandevia, 2021). However, when *p* values were below 0.10, we considered that value along with the calculated effect size (see next sentence) as well as the physiological relevance for each variable to draw conclusions about the result of a given comparison. Thus, we report effect sizes, where appropriate, for the primary data of interest to aid the reader with interpretations, where Cohen's *d* from 0.20 to 0.49 represents a small effect [slight], 0.50 to 0.79 represents a medium effect [moderate], and >0.80 represents a large effect (Cohen, 1988).

On an exploratory basis, we also examined the independent effects of several factors with the potential to influence pulmonary function values in adults with well‐healed burn injuries. We compared percent predicted pulmonary function values between adults with well‐healed burn injuries with a prior inhalation injury with adults with well‐healed burn injuries without an inhalation injury using unpaired, two‐tailed *t*‐tests. We also examined the strength of the association between percent predicted pulmonary function values and four variables, each independently, using simple linear regression: (1) Total body surface area burned as a percentage, (2) years since burn injury, (3) age at burn injury, and (4) thorax body surface area burned as a percentage.

## 3 RESULTS

### 3.1 Participants

We present participant demographics in Table 1. Medication usage is listed by medication class with the number [and proportion] of participants in the control group, followed by the number [and proportion] of participants in the burn‐injured group: Multivitamins/supplements (*n* = 6 [50%], *n* = 10 [24%]), for pain (*n* = 0, *n* = 8 [20%]), medical marijuana (*n* = 0, *n* = 6 [15%]), antihypertensive (*n* = 1 [8%], *n* = 4 [10%]), for hypothyroidism (*n* = 1 [8%], *n* = 3 [6%]), antidepressant (*n* = 0, *n *= 6 [15%]), hormonal birth control (*n* = 0, *n* = 5 [12%]), for allergies (*n* = 0, *n* = 4 [10%]), for hypercholesterolemia (*n* = 1 [8%], *n* = 2 [5%]), anti‐inflammatory (*n* = 0, *n* = 3 [7%]), stimulants (*n* = 2 [17%], *n* = 0), sedatives (*n* = 0, *n* = 3 [7%]), and other (*n* = 1 [8%], *n* = 11 [27%]). Lastly, two of 10 [20% of those evaluated] control participants and four of 26 adults with well‐healed burn injuries [15% of those evaluated] had a positive bronchodilator response.

**TABLE 1:** Participant screening information

Characteristic	Controls	Adults with well‐healed burn injuries	*p*	Cohen's *d*
Number of participants	12 (6 F / 6 M)	41 (20 F / 21 M)	–	–
Age, yrs	35 ± 8 (20–52)	40 ± 13 (21–60)	0.13	0.56
Body height, cm	173 ± 9 (162–189)	169 ± 9 (151–190)	0.24	0.40
Body mass, kg	84 ± 24 (39–122)	81 ± 19 (46–126)	0.67	0.13
Body mass index, kg/m^2^	29 ± 5 (20–38)	28 ± 6 (19–43)	0.59	0.18
Body surface area, m^2^	2.0 ± 0.3 (1.4–2.4)	1.9 ± 0.2 (1.4–2.5)	0.54	0.39
Body surface area burned, %	–	45 ± 20 (15–88)	–	–
Time since burn injury, yrs	–	17 ± 12 (2–50)	–	–

Note

We present data as mean ± SD with ranges. We compared group values using unpaired, two‐tailed *t*‐tests.

### 3.2 Spirometry

**FIGURE 1:**
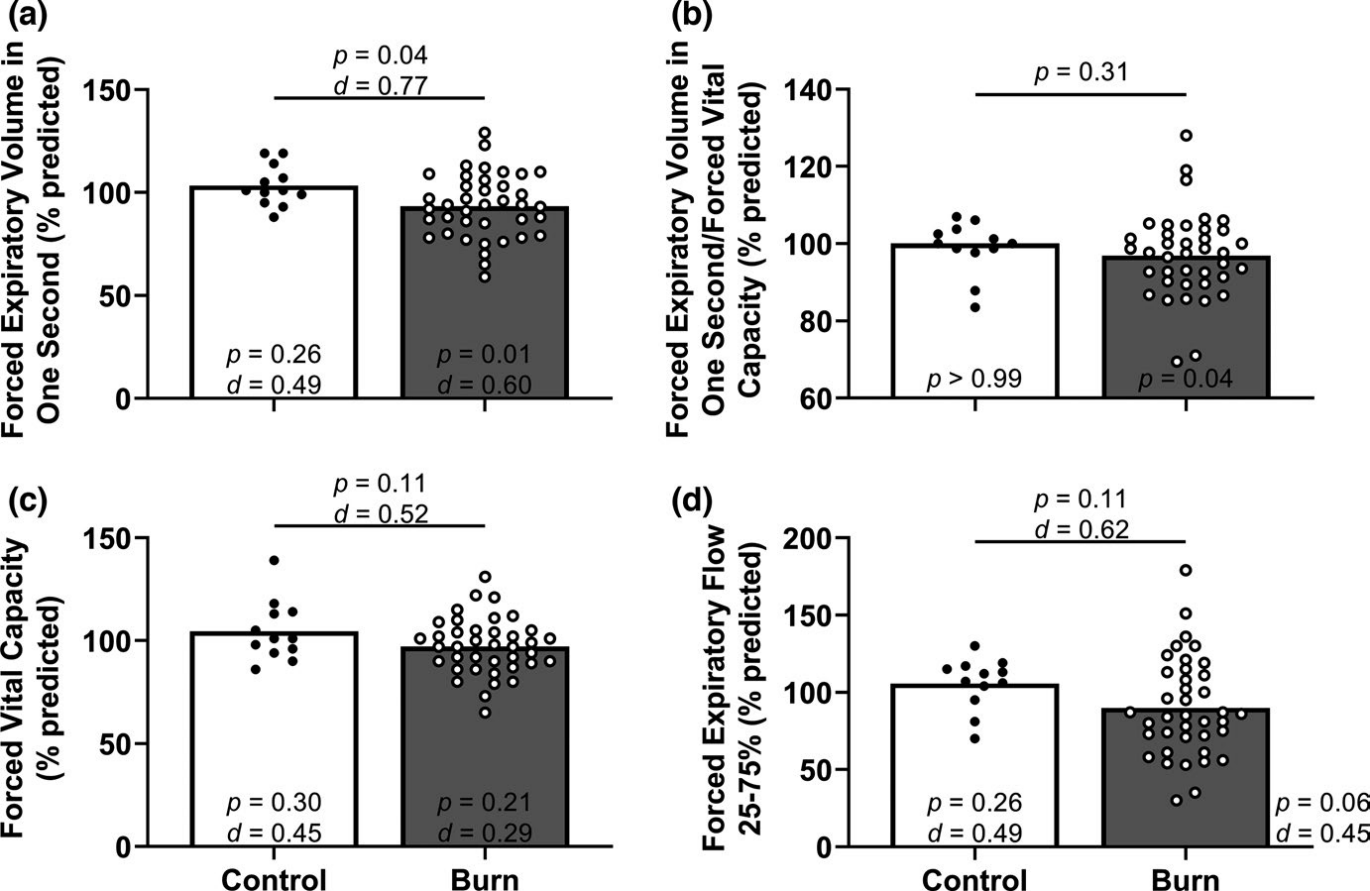
Spirometry. For percent predicted forced expiratory volume in one second/forced vital capacity (panel b), we compared group values using a Mann–Whitney test and present data as medians with individual values. For the other three panels, we compared group values using unpaired, two‐tailed *t*‐tests and present data as means with individual data. Finally, we compared the percent predicted forced expiratory volume in one second/forced vital capacity using Wilcoxon signed‐rank tests (theoretical median of 100), the other three other variables within each group using one‐sample, two‐tailed *t*‐tests (theoretical mean of 100) and present the results within (or directly aside) each bar. Control, control group of adults without a burn injury; Burn, adults with well‐healed burn injuries

Percent predicted FEV_1_ was moderately lower in adults with well‐healed burn injuries compared with the control group (Figure 1a). Percent predicted FVC, percent predicted FEV_1_/FVC, and percent predicted FEF_25–75%_ were not different between adults with well‐healed burn injuries and the control group (Figure 1b–d). Percent predicted FEV_1_ was moderately lower in adults with well‐healed burn injuries compared with the population mean (100% percent predicted; Figure 1a). Percent predicted FEF_25–75%_ was slightly lower in adults with well‐healed burn injuries compared with the population mean (Figure 1d). We also present the proportion of adults with well‐healed burn injuries and control adults who had values below the lower limit of normal for FVC (CON: 0/12, BURN: 2/39; *p* > 0.99, CI: 0.00–5.62), FEV_1_ (CON: 0/12, BURN: 7/39; *p* = 0.18, CI: 0.00–1.47), and FEF_25–75%_ (CON: 0/12, BURN: 3/39; *p* > 0.99, CI: 0.00–3.64). Absolute FEV_1_ and FEF_25–75%_ were lower in adults with well‐healed burn injuries (Table 2). Absolute FVC was moderately lower in adults with well‐healed burn injuries (Table 2).

**TABLE 2:** Absolute pulmonary function values

Measure	Controls	Adults with well‐healed burn injuries	*p*	Cohen's *d*
Forced expiratory volume in one second, L	3.9 ± 0.9 (2.8–5.6)	3.2 ± 0.8 (1.5–4.7)	<0.01	0.82
Forced vital capacity, L	4.9 ± 1.1 (3.4–7.0)	4.1 ± 1.0 (1.9–5.9)	0.03	0.76
Forced expiratory volume in one second/forced vital capacity, ratio (%)	82 ± 6 (71–92)	79 ± 10 (54–105)	0.42	0.36
Forced expiratory flow 25%–75%, L/s	4.1 ± 1.0 (2.7–5.5)	3.1 ± 1.2 (1.1–6.3)	<0.01	0.91
Maximal voluntary ventilation, L/min	146 ± 27 (107–195)	116 ± 34 (48–187)	<0.01	0.98
Total lung capacity, L	6.5 ± 1.3 (4.8–9.0)	5.9 ± 1.2 (3.5–9.1)	0.12	0.48
Residual volume/total lung capacity, ratio (%)	24 [21–28] (19–33)	28 [22–36] (17–47)	0.06	–
Vital capacity, L	5.0 ± 1.2 (3.5–7.1)	4.2 ± 0.9 (2.0–6.0)	0.02	0.75
Functional residual capacity, L	2.8 [2.3–3.3] (1.4–4.1)	2.6 [2.4–3.1] (1.6–5.7)	0.75	–
V_alv_, L	5.8 ± 1.3 (4.3–8.3)	5.1 ± 1.1 (2.6–7.9)	0.06	0.58
TL_CO_, mM/min/kPa	9.5 [9.0–11.0] (6.0–14.0)	7.7 [6.6–10.0] (5.1–13.0)	0.04	–
DL_CO_/V_alv_, ml/min/mmHg/L	5.2 [4.4–5.8] (4.0–6.4)	4.8 [4.5–5.4] (3.0–7.8)	0.24	–
Airway resistance, cmH_2_O/L/s	2.2 ± 0.6 (1.5–3.6)	2.8 ± 0.9 (1.1–5.7)	0.06	0.78
Specific airway resistance, cmH_2_O/L/s	7.4 ± 1.6 (5.3–9.7)	9.6 ± 3.4 (1.8–15.9)	0.03	0.83

Note

For TL_CO_ (Transfer Factor of the Lung for Carbon Monoxide), DL_CO_/V_alv_ (Diffusing Capacity of the Lung for Carbon Monoxide/Alveolar Volume), residual volume/total lung capacity, and functional residual capacity, we compared group values using Mann‐Whitney tests and present data as median & [IQR] with ranges. For all other variables, we compared group values using unpaired, two‐tailed *t*‐tests and present data as mean ± SD with ranges.

### 3.3 Maximal voluntary ventilation

**FIGURE 2:**
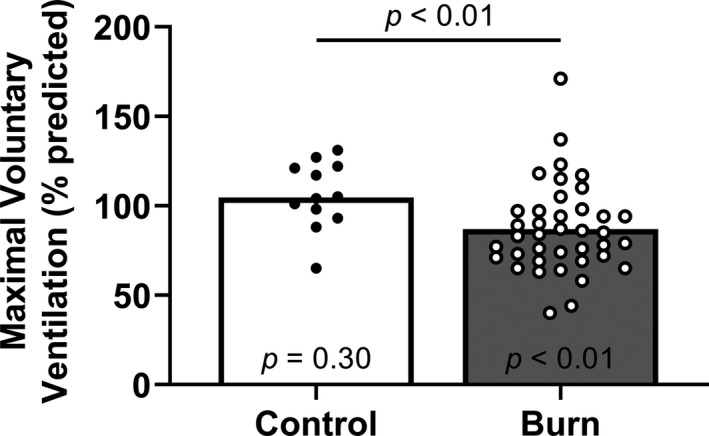
Maximal voluntary ventilation. We compared group values using a Mann–Whitney test and present data as medians with individual values. We also compared the percent predicted maximal voluntary ventilation within each group using Wilcoxon signed‐rank tests (theoretical median of 100) and present the results within each bar. Control, control group of adults without a burn injury; Burn, adults with well‐healed burn injuries

Percent predicted MVV was lower in adults with well‐healed burn injuries compared with the control group (Figure 2). Percent predicted MVV was lower in adults with well‐healed burn injuries compared with the population mean (Figure 2). Also, we present the proportion of adults with well‐healed burn injuries and control adults who had values below 80% of predicted MVV (CON: 1/12, BURN: 18/39; *p* = 0.02, CI: 0.01–0.79). Absolute MVV was lower in adults with well‐healed burn injuries (Table 2).

### 3.4 Lung volumes

**FIGURE 3:**
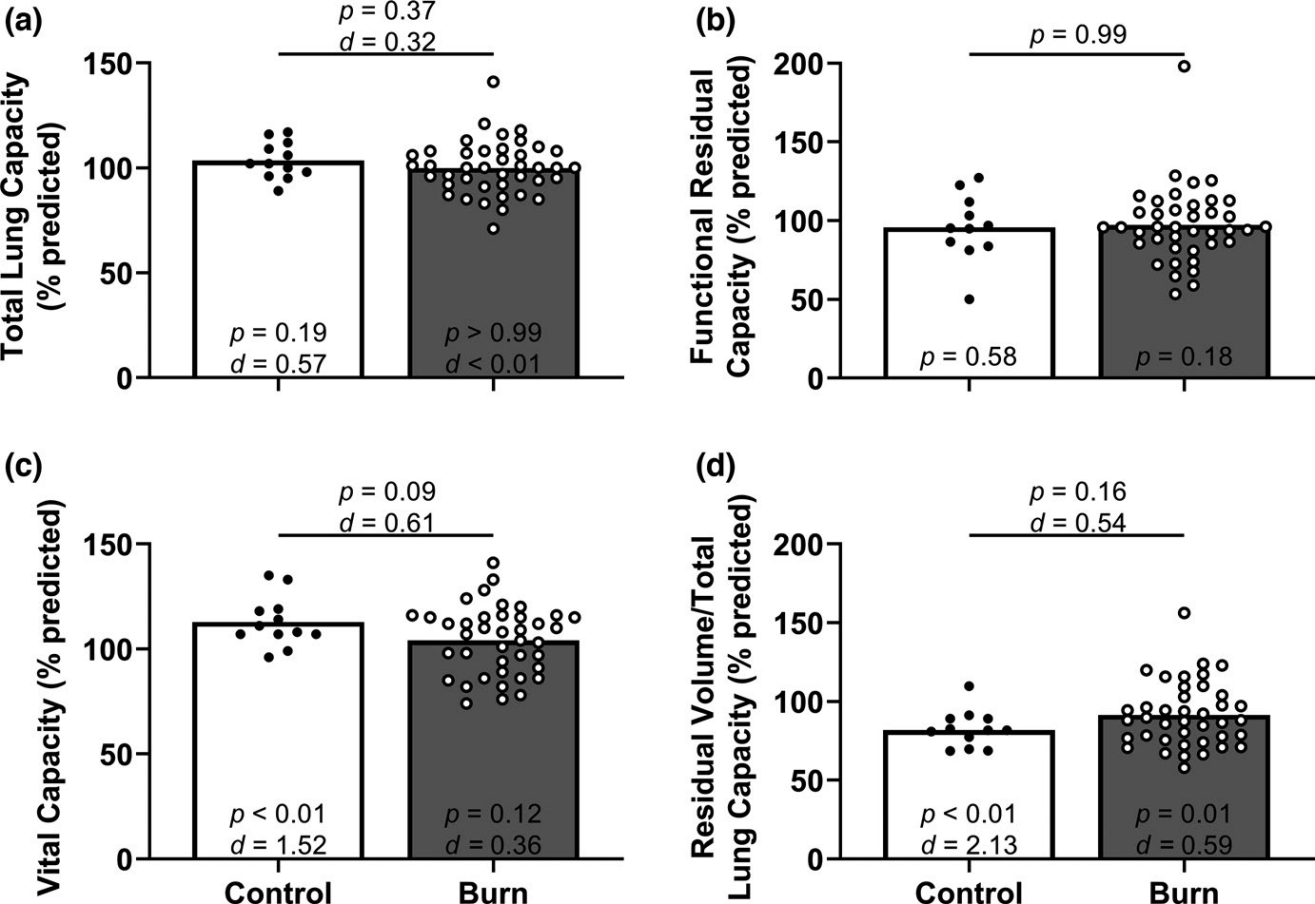
Lung volumes. For percent predicted functional residual capacity (panel b), we compared group values using a Mann–Whitney test and present data as medians with individual values. For the other three variables, we compared group values using unpaired, two‐tailed *t*‐tests and present data as means with individual data. Finally, we compared the percent predicted forced functional residual capacity using Wilcoxon signed‐rank tests (theoretical median of 100), the other three other variables within each group using one‐sample, two‐tailed *t*‐tests (theoretical mean of 100) and present the results within each bar. Control, control group of adults without a burn injury; Burn, adults with well‐healed burn injuries

Percent predicted TLC, percent predicted functional residual capacity, and percent predicted residual volume/TLC were not different between adults with well‐healed burn injuries and the non‐burned control group (Figure 3a,b,d). Percent predicted vital capacity was moderately lower in adults with well‐healed burn injuries compared with the non‐burned control group (Figure 3c). Percent predicted residual volume/total lung capacity was moderately lower in adults with well‐healed burn injuries compared with the population mean (Figure 3d). We also present the proportion of adults with well‐healed burn injuries and control adults who had values below 80% of predicted TLC (CON: 0/12, BURN: 1/40; *p* > 0.99, CI: 0.00–30.75). Absolute residual volume/TLC was moderately higher in adults with well‐healed burn injuries (Table 2). Absolute vital capacity and alveolar volume (V_alv_) were moderately lower in adults with well‐healed burn injuries (Table 2).

### 3.5 Lung diffusing capacity

**FIGURE 4:**
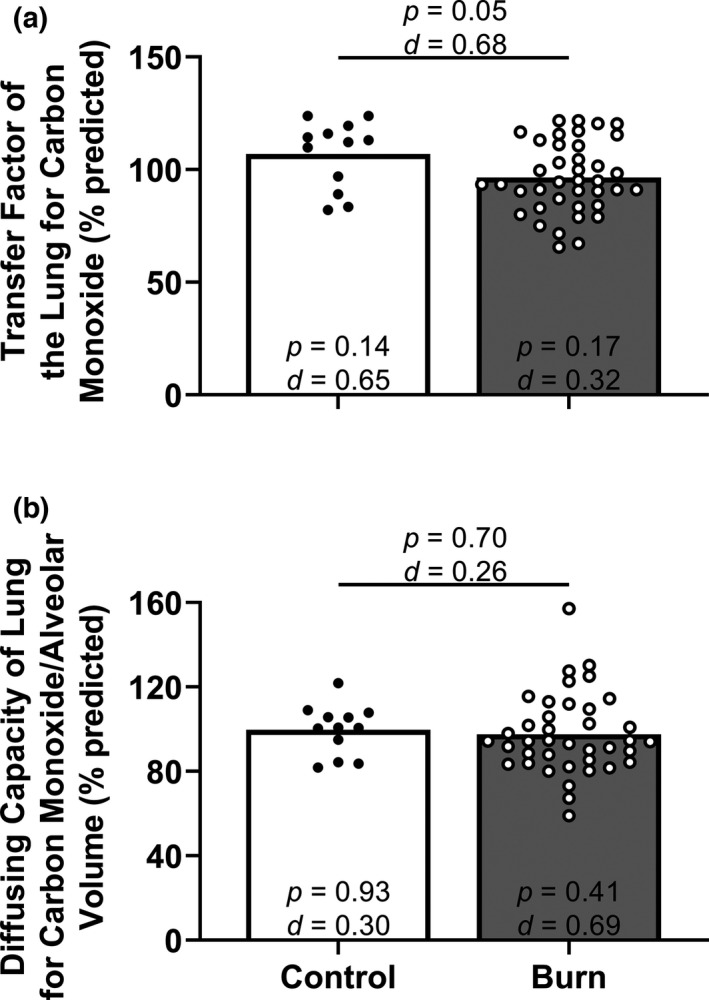
Lung diffusing capacity. We compared group values using unpaired, two‐tailed *t*‐tests and present data as means with individual data. We also compared the percent predicted values within each group using one‐sample, two‐tailed *t*‐tests (theoretical mean of 100) and present the results within each bar. Control, control group of adults without a burn injury; Burn, adults with well‐healed burn injuries

Percent predicted TL_CO_ was moderately lower in adults with well‐healed burn injuries compared with the control group (Figure 4a). This result was maintained following hemoglobin concentration adjustment for TL_CO_ (between groups *t*‐test: *p* = 0.04, Cohen's *d* = 0.71; one‐sample *t*‐test for Control versus 100% predicted: *p* = 0.14, Cohen's *d* = 0.66, one‐sample *t*‐test for Burn versus 100% predicted: *p* = 0.14, Cohen's *d* = 0.34; data not shown). Percent predicted DL_CO_/V_alv_ was not different between adults with well‐healed burn injuries compared with the control group (Figure 4b). Additionally, we present the proportion of adults with well‐healed burn injuries and control adults who had values below the lower limit of normal for TL_CO_ (CON: 0/12, BURN: 4/39; *p* = 0.56, CI: 0.00–2.67) and below 80% of predicted DL_CO_/V_alv_ (CON: 0/12, BURN: 3/39; *p* > 0.99, CI: 0.00–3.78). Absolute TL_CO_ was lower in adults with well‐healed burn injuries (Table 2). Absolute DL_CO_/V_alv_ was not different between groups (Table 2).

### 3.6 Airway resistance

**FIGURE 5:**
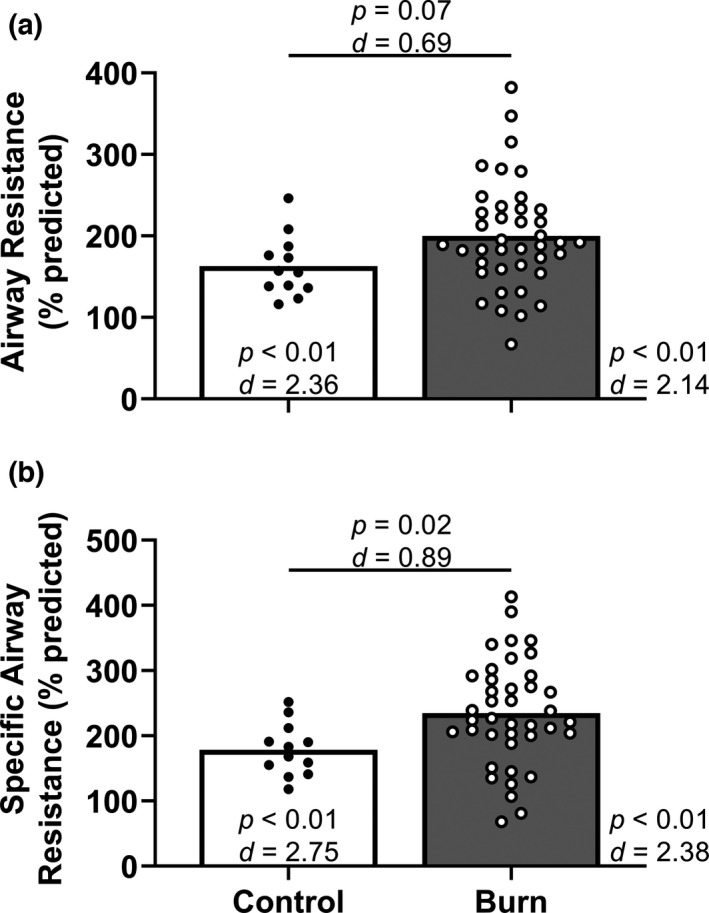
Airway resistance. We compared group values using unpaired, two‐tailed *t*‐tests and present data as means with individual data. We also compared the percent predicted values within each group using one‐sample, two‐tailed *t*‐tests (theoretical mean of 100) or Wilcoxon signed‐rank tests (theoretically median of 100) and present the results within (or directly aside) each bar. Control, control group of adults without a burn injury; Burn, adults with well‐healed burn injuries

Percent predicted airway resistance was moderately higher in adults with well‐healed burn injuries (*n* = 40) compared with the non‐burned control group (Figure 5a). Specific airway resistance (adjusted for thoracic gas volume) was higher in adults with well‐healed burn injuries (*n* = 40) compared with the control group (Figure 5b). Percent predicted airway resistance and specific airway resistance were also higher in adults with well‐healed burn injuries when compared with the population mean (Figure 5a,b). Absolute specific airway resistance was higher in adults with well‐healed burn injuries (Table 2). Absolute airway resistance was moderately higher in adults with well‐healed burn injuries (Table 2).

### 3.7 Independent effects of inhalation injury, total burn coverage, time since injury, age at injury, and thorax burn coverage

The percent predicted TLC was higher in adults with well‐healed burn injuries with (*n* = 28) an inhalation injury compared with adults with well‐healed burn injuries without (*n* = 13) an inhalation injury (Table 3). All other comparisons resulted in no or only slight differences between adults with well‐healed burn injuries with an inhalation injury and adults with well‐healed burn injuries without an inhalation injury (Table 3). Body surface area size of the burn injury and time since the burn injury were each not meaningfully related to any percent predicted pulmonary function value (Table 3). Finally, age at burn injury (*R* ^2^ ≤ 0.02, *p* ≥ 0.43 for all; data not shown) and thorax burn coverage (*R* ^2^ ≤ 0.05, *p* ≥ 0.21 for all; data not shown) were not meaningfully related to any percent predicted pulmonary function value.

**TABLE 3:** Influence of inhalation injury history, size of body surface area burned, and time since injury on key variables

	Inhalation injury	Body surface area burned	Time since injury
Percent predicted measure	Cohen's *d* (*p*)	*R* ^2^ *(p)*	*R* ^2^ *(p)*
Forced expiratory volume in one second	0.37 (0.31)	0.06 (0.15)	<0.01 (0.83)
Forced vital capacity	0.14 (0.69)	0.08 (0.08)	<0.01 (0.95)
Forced expiratory volume in one second/forced vital capacity	0.22 (0.56)	<0.01 (0.78)	<0.01 (0.81)
Forced expiratory flow 25%–75%	– (0.55)	<0.01 (0.66)	<0.01 (0.67)
Maximal voluntary ventilation	0.45 (0.19)	<0.01 (0.99)	0.04 (0.22)
Total lung capacity	– (0.03)^a^	0.02 (0.45)	<0.01 (0.94)
Residual volume/total lung capacity	– (0.46)	0.02 (0.86)	0.04 (0.22)
Vital capacity	– (0.53)	0.07 (0.10)	<0.01 (0.62)
Functional residual capacity	– (0.72)	<0.01 (0.61)	<0.01 (0.32)
TL_CO_	0.45 (0.24)	<0.01 (0.76)	0.02 (0.43)
DL_CO_/V_alv_	0.44 (0.22)	0.10 (0.05)	0.09 (0.07)
Airway resistance	0.04 (0.92)	<0.01 (0.98)	0.02 (0.41)
Specific airway resistance	0.46 (0.21)	<0.01 (0.99)	0.01 (0.46)

Note

Inhalation injury: For forced expiratory flow 25%–75%, total lung capacity, residual volume/total lung capacity, vital capacity, and functional residual capacity (all percent predicted values), we compared groups using Mann‐Whitney tests. For all other variables, we compared groups using unpaired, two‐tailed *t*‐tests.

Body surface area burned & years after injury: We used simple linear regression for all variables. TL_CO_, Transfer Factor of the Lung for Carbon Monoxide; DL_CO_/V_alv_, diffusing Capacity of the Lung for Carbon Monoxide/Alveolar Volume.

^a^ Indicates that the median value for burn‐injured adults with an inhalation injury was greater than the median value for burn‐injured adults without an inhalation injury.

## 4 DISCUSSION

The purpose of our study was to test the hypothesis that adults with well‐healed burn injuries have lower pulmonary function values than adults without a prior burn injury. Our discussion will focus on the percent predicted values obtained because these values are adjusted for information (e.g., age, body height, sex, and race/ethnicity in prediction equations) that influence comparisons to adults without prior burn injuries, who were also tested within this study. Our primary finding is that adults with well‐healed burn injuries had lower FEV_1_, FEF_25–75%_, and MVV, as well as higher airway resistance, when compared with the theoretical population mean of 100 for percent predicted values. Also, we found that adults with well‐healed burn injuries have a lower percent predicted FEV_1_, MVV, TL_CO_, as well as greater airway resistance and specific airway resistance values when compared with the non‐burned control group. It is notable that some proportion of adults with well‐healed burn injuries did not have lower pulmonary function values. Our exploratory analyses suggest that such deficits in pulmonary function among adults with well‐healed burn injuries are not strongly related to a history of an inhalation injury, total burn coverage, the time that has passed since the injury occurred, the age at which an individual sustained the injury, or thorax burn coverage. Together, these data—the most comprehensive assessment of pulmonary function among adults with well‐healed burn injuries—emphasize an impairment that requires attention in the long‐term rehabilitation of this clinical population, particularly in those with the lowest pulmonary function values.

Previous studies report conflicting findings regarding whether FEV_1_/FVC and/or MVV is reduced in adults with well‐healed burn injuries (Grisbrook et al., 2012; Özkal et al., 2021; Willis et al., 2011; Won et al., 2021). We did not find FEV_1_/FVC to be lower in our cohort, which is consistent with some (Özkal et al., 2021; Willis et al., 2011; Won et al., 2021), but not all (Grisbrook et al., 2012) prior work. Similarly, we found that MVV was lower in adults with well‐healed burn injuries, which is consistent with some (Willis et al., 2011; Won et al., 2021), but not all (Grisbrook et al., 2012) prior work. Such discrepancies from our findings and those from Grisbrook et al. (2012) could be due to the cohort selection, as Grisbrook et al. recruited adults who “were still experiencing a functional deficit that persisted following standard rehabilitation,” whereas the individuals recruited for the present work were not recruited to meet this criterion. However, this conclusion is speculative and future work is needed to better parse out these concerns. When taken together, our data advance an understanding of the deficits in pulmonary function among adults with well‐healed burn injuries.

These are the first data to demonstrate that adults with well‐healed burn injuries have high airway resistance and specific airway resistance (adjusted for thoracic gas volume (Kaminsky, 2012)). We also found that TL_CO_ was moderately low in adults with well‐healed burn injuries. This finding is consistent with prior reports that mean values for DL_CO_ were 76%–81% of predicted—that is, well below the theoretical mean of 100% (Won et al., 2020, 2021). However, others (Cerfolio & Bryant, 2009) contend that DL_CO_/V_alv_ (adjusted for alveolar volume) provides more clinical insight regarding an individual's capability to exchange gases for a given surface area available for gas exchange as well as insight into the integrity of the alveolar‐capillary interface. Therefore, our finding of no group differences for DLCO/V_alv_ (adjusted for alveolar volume) suggests that adults with well‐healed burn injuries do not have lower pulmonary gas exchange capabilities.

### 4.1 Inhalation injuries

In children, pulmonary findings suggestive of restrictive and/or obstructive disorders were more common in those who had experienced an inhalation injury compared with those without such an injury (Desai et al., 1993). In adults with burn injuries assessed at discharge, the presence of an inhalation injury was associated with a lower FEV_1_/FVC (Özkal et al., 2021). In adults with burn injuries assessed three months after injury, those with an inhalation injury had a lower FEV_1_ and FVC (Won et al., 2021). However, a retrospective assessment of long‐term lung function in patients with bronchoscopic evidence of smoke inhalation showed that combined inhalation injury and body surface burn do not necessarily imply long‐term damage to the respiratory airways or lungs (Bourbeau et al., 1996). These reports in humans mirror data in animals demonstrating that smoke inhalation elicits direct alveolar damage (potentially via inflammation), alveolar blood flow obstruction due to edema, and increases in pulmonary vascular resistance resulting in increased physiological dead space (Stephenson et al., 1975). Further, lung compliance is reduced (i.e., the lung stiffens) via interstitial edema, decreased surfactant, and some degree of atelectasis and/or bronchospasm (Stephenson et al., 1975). Despite prior evidence of deleterious effects of inhalation injury in humans and animals, we found that total lung capacity was greater among those with a prior inhalation injury compared with those without an inhalation injury, which was contrary to our hypothesis. Additionally, all other evaluated variables (see Table 3) were not meaningfully lower in those with a prior inhalation injury compared with those without an inhalation injury. One reason for a discrepancy between prior work and current work is the potential recovery of inhalation injury‐related deficits in pulmonary function, which may partially occur about five months after injury (Whitener et al., 1980). Nonetheless, future work is necessary to determine the extent to which a prior inhalation injury affects pulmonary function, given that the present work was an exploratory analysis using data from a larger study that aimed to address a secondary hypothesis. Therefore, we have limited information regarding the severity of the inhalation injury sustained among our cohort of adults with well‐healed burn injuries who were studied as far out as 50 years post‐injury.

### 4.2 Body surface area burned

Because larger burn injuries produce a more profound insult (e.g., hypermetabolism, as discussed below), requiring longer bed rest, we reasoned that those with a higher body surface area burn‐injured would have a more compromised pulmonary function. In support of this, a larger body surface area burned is associated with longer bed rest, which is notable as bed rest alone impairs physiological function (e.g., respiratory muscle weakness) (Saltin et al., 1968; Whitener et al., 1980). Moreover, a recent study reported that total body surface area burned was moderately related to lower FEV_1_ and FVC at hospital discharge (Özkal et al., 2021). To our surprise, there was no meaningful relation between the percentage of body surface area burned and values for any pulmonary function measure in our cohort tested years‐to‐decades after recovery. It is unclear why a larger burn surface area was not associated with worsened pulmonary function. We speculate that there is a ceiling effect whereby even those with the lowest burn coverages in our cohort were likely hospitalized for multiple weeks, which could have elicited irreversible damage that would not necessarily be worsened by additional weeks/months in such a setting. Indeed, prior work supports this notion with the finding that adults with body surface area burn coverages of 20%–40% have a very robust pathophysiological response that is similar to larger burn coverages (Stanojcic et al., 2018). Moreover, prior work suggests that individuals with burn injuries on the chest wall do not have lower pulmonary function relative to individuals with burn injuries in other anatomical locations (Won et al., 2021). In agreement, we found that thorax burn coverage was not meaningfully associated with any pulmonary function value. Nonetheless, future studies are warranted to confirm or refute the findings that a larger burn coverage is associated with greater impairments in pulmonary function.

### 4.3 Time since injury

Previous work demonstrates that hypermetabolism and catabolism continue for at least nine months among children who survived severe burn injuries (Hart et al., 2000). Moreover, resting energy expenditure and inflammatory cytokines may be elevated for 24 months after a burn injury (Jeschke et al., 2011). However, the timeline of pulmonary function recovery in adults with well‐healed burn injuries remains unknown. Our cohort of individuals (tested at least 2 years post‐burn injury and up to 50 years post‐burn injury) did not show any obvious trends for the relation between pulmonary function and the time since the injury (see Table 3). Thus, our data support the possibility that any reductions in pulmonary function that occur within the first two years after a burn injury are not improved in the following decades. It is important to note, however, that such speculation must be confirmed with longitudinal data among adults with well‐healed burn injuries. Lastly, our findings of lower pulmonary function are consistent with findings among children (Duke et al., 2016) and an appreciably smaller cohort of adults with well‐healed burn injuries (Mlcak et al., 1998) several years after injury.

### 4.4 Clinical significance and future directions

We found that adults with well‐healed burn injuries had a lower percent of predicted FEV_1_, a lower percent predicted MVV, and a higher percent predicted specific airway resistance than non‐burned control participants. None of these variables were explained by having a previous inhalation injury, the size of the body surface area burned, or the time since the burn injury. Together, these data suggest that many adults with well‐healed burn injuries continue to have lower pulmonary function decades after injury. However, longitudinal studies are warranted to determine if a prior burn injury influences the slope of the age‐related reductions in pulmonary function values. Separately, and importantly, lower MVV could provoke changes in breathing mechanics during exercise (Babb, 2013). Specifically, these changes could increase the risk of airflow limitation, dynamic hyperinflation, and alterations in breathing pattern; all of which could increase the work of breathing and impose a ceiling on maximal ventilation (a ventilatory limitation to activity). Further, such responses could contribute to exacerbated exertional dyspnea sensations. Therefore, future research should examine potential rehabilitation strategies that could improve pulmonary function in adults with well‐healed burn injuries, particularly in those with the lowest pulmonary function values.

Twelve weeks of exercise training following a burn injury in children aged 7–17 years old improved FVC, FEV_1_, and MVV (Suman et al., 2002). Such a finding was not observed in a small study of adults with well‐healed burn injuries following 12 weeks of exercise training (Grisbrook et al., 2012). In the study on children (Suman et al., 2002), FVC, FEV_1_, and MVV were remarkably reduced pre‐training in the patient population as compared with controls. It could be that whole‐body and/or respiratory muscle exercise training increases respiratory muscle strength as well as the ability to expand the lungs and chest wall, which would increase all three aforementioned variables. Indeed, recent work shows promise for inspiratory muscle strength training to improve respiratory muscle strength among adults with well‐healed burn injuries (Abazarnejad et al., 2021). Such an intervention may be a useful alternative to traditional exercise training among adults with well‐healed burn injuries, a group with disproportionately low cardiorespiratory fitness and physical activity levels (Dodd et al., 2017; Duke et al., 2017; Ferrando et al., 1997; Ganio et al., 2015; Willis et al., 2011). Thus, future work is necessary to identify and optimize traditional (e.g., exercise) and novel (e.g., respiratory muscle strength training) strategies to improve pulmonary function among adults with well‐healed burn injuries years after their injury.

### 4.5 Limitations

First, while some (Desai et al., 1993; Mlcak et al., 1995), but not all (Rivas et al., 2018), previous work has demonstrated a greater pulmonary dead space in children with prior burn injuries, we did not discuss physiological dead space in the present manuscript because it was not assessed. Additionally, these data represent a secondary aim of a larger project and as noted above, future studies specifically designed to address such questions are needed to confirm our observations. Related to the fact that these data address a secondary research question of a larger project, we do not have complete smoking history data available for all participants, particularly in the control group. In the individuals with well‐healed burn injuries, five adults reported a smoking history. Of the four adults that reported further details for their smoking history, there was a mean and standard deviation of 5.4 ± 4.9 pack‐years. The sub‐group of prior smokers (*n* = 5) did not differ from the subgroup of non‐smokers (*n* = 36) among burn‐injury survivors for key variables (*p *> 0.36 for percent predicted FEV1, percent predicted MVV, and percent predicted sRaw). Lastly, bronchodilator responsiveness was not assessed in all participants. Therefore, we cannot rule out the presence of an obstructive airway disorder in some of the participants in this cohort. However, the sub‐group of adults with well‐healed burn injuries with a positive bronchodilator response (*n* = 4) did not differ from the other 37 adults with well‐healed burn injuries for key variables (*p *> 0.28 for percent predicted FEV1, percent predicted MVV, and percent predicted sRaw). Future studies are warranted to fill knowledge gaps that remain from the present work.

## 5 CONCLUSIONS

In summary, we found that adults with well‐healed burn injuries had a lower percentage of predicted forced expiratory volume in one second, a lower percent predicted maximal voluntary ventilation, and a higher percent predicted specific airway resistance than control participants without a prior burn injury. None of these variables were explained by having a previous inhalation injury, having a larger portion of body surface area burned, or a shorter time since the burn injury. Together, these data suggest that adults with well‐healed burn injuries continue to have lower pulmonary function decades after injury. Therefore, future research should examine potential rehabilitation strategies that could improve pulmonary function among adults with well‐healed burn injuries.

## CONFLICT OF INTEREST

The authors have no competing interests to declare.

## AUTHOR CONTRIBUTIONS

CGC and TGB conceived the research project. All authors performed acquisition, analysis, and/or interpretation of data. JCW and CGC drafted the manuscript. All authors revised it critically for important intellectual content and approve the final version to be submitted.

## References

[phy215264-bib-0001] Abazarnejad, E. , Froutan, R. , Ahmadabadi, A. , & Mazlom, S. R. (2021). Improving respiratory muscle strength and health status in burn patients: a randomized controlled trial. Quality of Life Research, 31(3), 769–776. 10.1007/s11136-021-02996-x 34535839

[phy215264-bib-0002] Babb, T. G. (2013). Exercise ventilatory limitation: the role of expiratory flow limitation. Exercise and Sport Sciences Reviews, 41, 11–18. 23038244 10.1097/JES.0b013e318267c0d2 PMC3529766

[phy215264-bib-0003] Bourbeau, J. , Lacasse, Y. , Rouleau, M. Y. , & Boucher, S. (1996). Combined smoke inhalation and body surface burns injury does not necessarily imply long‐term respiratory health consequences. European Respiratory Journal, 9, 1470–1474. 10.1183/09031936.96.09071470 8836661

[phy215264-bib-0004] Burrows, B. , Kasik, J. E. , Niden, A. H. , & Barclay, W. R. (1961). Clinical usefulness of the single‐breath pulmonucy diffusing capacity test. American Review of Respiratory Disease, 84, 789–806. 13875021 10.1164/arrd.1961.84.6.789

[phy215264-bib-0005] Cerfolio, R. J. , & Bryant, A. S. (2009). Different diffusing capacity of the lung for carbon monoxide as predictors of respiratory morbidity. The Annals of Thoracic Surgery, 88(2), 405–411. 19632384 10.1016/j.athoracsur.2009.04.015

[phy215264-bib-0006] Cohen, J. (1988). Statistical Power Analysis for the Behavioral Sciences. Routledge.

[phy215264-bib-0007] Curran‐Everett, D. (2020). Evolution in statistics: P values, statistical significance, kayaks, and walking trees. Advances in Physiology Education, 44, 221–224. 32412384 10.1152/advan.00054.2020

[phy215264-bib-0008] Desai, M. H. , Micak, R. P. , Robinson, E. , McCauley, R. L. , Carp, S. S. , Robson, M. C. , & Herndon, D. N. (1993). Does inhalation injury limit exercise endurance in children convalescing from thermal injury? The Journal of Burn Care & Rehabilitation, 14, 12–16. 10.1097/00004630-199301000-00004 8454658

[phy215264-bib-0009] Peck, M. D. (2011). Epidemiology of burns throughout the world. Part I: Distribution and risk factors. Burns, 37, 1087–1100. 21802856 10.1016/j.burns.2011.06.005

[phy215264-bib-0010] Dodd, H. , Fletchall, S. , Starnes, C. , & Jacobson, K. (2017). Current concepts burn rehabilitation, Part II: Long‐term recovery. Clinics in Plastic Surgery, 44, 713–728. 28888297 10.1016/j.cps.2017.05.013

[phy215264-bib-0011] Dubois, A. B. , Botelho, S. Y. , & Comroe, J. H. Jr (1956). A new method for measuring airway resistance in man using a body plethysmograph: Values in normal subjects and in patients with respiratory disease. Journal of Clinical Investigation, 35, 327–335. 13295397 10.1172/JCI103282 PMC438815

[phy215264-bib-0012] Duke, J. M. , Boyd, J. H. , Randall, S. M. , & Wood, F. M. (2015). Long term mortality in a population‐based cohort of adolescents, and young and middle‐aged adults with burn injury in Western Australia: A 33‐year study. Accident Analysis and Prevention, 85, 118–124. 26432064 10.1016/j.aap.2015.09.011

[phy215264-bib-0013] Duke, J. M. , Randall, S. M. , Fear, M. W. , Boyd, J. H. , Rea, S. , & Wood, F. M. (2016). Respiratory morbidity after childhood burns: A 10‐year follow‐up study. Pediatrics, 138. 10.1542/peds.2016-1658 27664086

[phy215264-bib-0014] Duke, J. M. , Randall, S. M. , Fear, M. W. , O'Halloran, E. , Boyd, J. H. , Rea, S. , & Wood, F. M. (2017). Long term cardiovascular impacts after burn and non‐burn trauma: A comparative population‐based study. Burns, 43, 1662–1672. 29032972 10.1016/j.burns.2017.08.001

[phy215264-bib-0015] Ferrando, A. A. , Tipton, K. D. , Bamman, M. M. , & Wolfe, R. R. (1997). Resistance exercise maintains skeletal muscle protein synthesis during bed rest. Journal of Applied Physiology, 82, 807–810. 10.1152/jappl.1997.82.3.807 9074967

[phy215264-bib-0016] Gandevia, S. (2021). Publications, replication and statistics in physiology plus two neglected curves. Journal of Physiology, 599, 1719–1721. 10.1113/JP281360 33507571

[phy215264-bib-0017] Ganio, M. S. , Pearson, J. , Schlader, Z. J. , Brothers, R. M. , Lucas, R. A. , Rivas, E. , Kowalske, K. J. , & Crandall, C. G. (2015). Aerobic fitness is disproportionately low in adult burn survivors years after injury. Journal of Burn Care & Research, 36, 513–519. 10.1097/BCR.0b013e3182a22915 24043241 PMC3954961

[phy215264-bib-0018] Goldman, H. I. , & Becklake, M. R. (1959). Respiratory function tests; normal values at median altitudes and the prediction of normal results. American Review of Tuberculosis, 79, 457–467. 13650117 10.1164/artpd.1959.79.4.457

[phy215264-bib-0019] Graham, B. L. , Brusasco, V. , Burgos, F. , Cooper, B. G. , Jensen, R. , Kendrick, A. , MacIntyre, N. R. , Thompson, B. R. , & Wanger, J. (2017). ERS/ATS standards for single‐breath carbon monoxide uptake in the lung. European Respiratory Journal, 49, 1600016. 10.1183/13993003.00016-2016 28049168

[phy215264-bib-0020] Graham, B. L. , Steenbruggen, I. , Miller, M. R. , Barjaktarevic, I. Z. , Cooper, B. G. , Hall, G. L. , Hallstrand, T. S. , Kaminsky, D. A. , McCarthy, K. , McCormack, M. C. , Oropez, C. E. , Rosenfeld, M. , Stanojevic, S. , Swanney, M. P. , & Thompson, B. R. (2019). Standardization of spirometry 2019 update. An Official American Thoracic Society and European Respiratory Society Technical Statement. American Journal of Respiratory and Critical Care Medicine, 200, e70–e88. 10.1164/rccm.201908-1590ST 31613151 PMC6794117

[phy215264-bib-0021] Grimby, G. , & Soderholm, B. (1963). Spirometric studies in normal subjects: III. Static lung volumes and maximum voluntary ventilation in adults with a note on physical fitness 1. Acta Medica Scandinavica, 173, 199–206. 13970718

[phy215264-bib-0022] Grisbrook, T. L. , Wallman, K. E. , Elliott, C. M. , Wood, F. M. , Edgar, D. W. , & Reid, S. L. (2012). The effect of exercise training on pulmonary function and aerobic capacity in adults with burn. Burns, 38, 607–613. 10.1016/j.burns.2011.11.004 22342175

[phy215264-bib-0023] Hankinson, J. L. , Odencrantz, J. R. , & Fedan, K. B. (1999). Spirometric reference values from a sample of the general U.S. population. American Journal of Respiratory and Critical Care Medicine, 159, 179–187. 10.1164/ajrccm.159.1.9712108 9872837

[phy215264-bib-0024] Hart, D. , Wolf, S. , Mlcak, R. , Chinkes, D. , Ramzy, P. , Obeng, M. , Ferrando, A. , Wolfe, R. , & Herndon, D. (2000). Persistence of muscle catabolism after severe burn. Surgery, 128, 312–319. 10.1067/msy.2000.108059 10923010

[phy215264-bib-0025] Hollowell, J. G. , van Assendelft, O. W. , Gunter, E. W. , Lewis, B. G. , Najjar, M. , & Pfeiffer, C. (2005) Hematological and iron‐related analytes‐‐reference data for persons aged 1 year and over: United States, 1988‐94. Vital and health statistics Series 11, Data from the national health survey 1‐156. 15782774

[phy215264-bib-0026] Huang, M. , Moralez, G. , Romero, S. A. , Jaffery, M. F. , Cramer, M. N. , Petric, J. K. , Nabasny, A. D. , & Crandall, C. G. (2020). The benefits of an unsupervised exercise program in persons with well‐healed burn injuries within the International Classification of Functioning, Disability and Health (ICF). Burns, 46, 1280–1288. 10.1016/j.burns.2020.06.023 32660830 PMC7529932

[phy215264-bib-0027] Jeschke, M. G. , Chinkes, D. L. , Finnerty, C. C. , Kulp, G. , Suman, O. E. , Norbury, W. B. , Branski, L. K. , Gauglitz, G. G. , Mlcak, R. P. , & Herndon, D. N. (2008). The pathophysiologic response to severe burn injury. Annals of Surgery, 248, 387–401. 10.1097/SLA.0b013e3181856241 18791359 PMC3905467

[phy215264-bib-0028] Jeschke, M. G. , Gauglitz, G. G. , Kulp, G. A. , Finnerty, C. C. , Williams, F. N. , Kraft, R. , Suman, O. E. , Mlcak, R. P. , & Herndon, D. N. (2011). Long‐term persistance of the pathophysiologic response to severe burn injury. PLoS One, 6, e21245. 10.1371/journal.pone.0021245 21789167 PMC3138751

[phy215264-bib-0029] Kaminsky, D. A. (2012). What does airway resistance tell us about lung function? Respiratory Care, 57(1), 85–99. 22222128 10.4187/respcare.01411

[phy215264-bib-0030] Kory, R. C. , Callahan, R. , Boren, H. G. , & Syner, J. C. (1961). The veterans administration‐army cooperative study of pulmonary function. American Journal of Medicine, 30, 243–258. 10.1016/0002-9343(61)90096-1 13753281

[phy215264-bib-0031] Mlcak, R. , Desai, M. , Robinson, E. , McCauley, R. , Richardson, J. , & Herndon, D. (1995). Increased physiological dead space/tidal volume ratio during exercise in burned children. Burns, 21, 337–339. 10.1016/0305-4179(94)00017-4 7546253

[phy215264-bib-0032] Mlcak, R. , Desai, M. H. , Robinson, E. , Nichols, R. , & Herndon, D. N. (1998). Lung function following thermal injury in children–an 8‐year follow up. Burns, 24, 213–216. 10.1016/S0305-4179(98)00012-6 9677023

[phy215264-bib-0033] Nylen, E. , Jeng, J. , Jordan, M. , Snider, R. , Thompson, K. , Lewis, M. , O'Neill, W. , & Becker, K. (1995). Late pulmonary sequela following burns: persistence of hyperprocalcitonemia using a 1–57 amino acid N‐terminal flanking peptide assay. Respiratory Medicine, 89, 41–46. 7708979 10.1016/0954-6111(95)90069-1

[phy215264-bib-0034] Özkal, Ö. , Topuz, S. , Karahan, S. , Erdem, M. M. , Konan, A. , & Yastı, A. (2021). Clinical predictors of pulmonary functions, respiratory/peripheral muscle strength and exercise capacity at discharge in adults with burn injury. Disability and Rehabilitation, 43, 2875–2881. 10.1080/09638288.2020.1720320 31999499

[phy215264-bib-0035] Pavoni, V. , Gianesello, L. , Paparella, L. , Buoninsegni, L. T. , & Barboni, E. (2010). Outcome predictors and quality of life of severe burn patients admitted to intensive care unit. Scandinavian Journal of Trauma, Resuscitation and Emergency Medicine, 18, 24. 10.1186/1757-7241-18-24 20420719 PMC2873368

[phy215264-bib-0036] Porter, C. , Hardee, J. P. , Herndon, D. N. , & Suman, O. E. (2015). The role of exercise in the rehabilitation of patients with severe burns. Exercise and Sport Sciences Reviews, 43, 34–40. 10.1249/JES.0000000000000029 25390300 PMC4272612

[phy215264-bib-0037] Rivas, E. , Herndon, D. N. , Cambiaso‐Daniel, J. , Rontoyanni, V. G. , Porter, C. , Glover, S. , & Suman, O. E. (2018). Quantification of an exercise rehabilitation program for severely burned children: The standard of care at shriners hospitals for children^®^‐galveston. Journal of Burn Care & Research, 39, 889–896. 29596648 10.1093/jbcr/iry001 PMC6060021

[phy215264-bib-0038] Romero, S. A. , Moralez, G. , Jaffery, M. F. , Huang, M. , Cramer, M. N. , Romain, N. , Kouda, K. , Haller, R. G. , & Crandall, C. G. (2019). Progressive exercise training improves maximal aerobic capacity in individuals with well‐healed burn injuries. American Journal of Physiology: Regulatory, Integrative and Comparative Physiology, 317, R563–r570. 10.1152/ajpregu.00201.2019 31433672 PMC6842906

[phy215264-bib-0039] Saltin, B. , Blomqvist, G. , Mitchell, J. H. , Johnson, R. L. Jr , Wildenthal, K. , & Chapman, C. B. (1968). Response to exercise after bed rest and after training. Circulation, 38, Vii1‐78. 5696236

[phy215264-bib-0040] Stanojcic, M. , Abdullahi, A. , Rehou, S. , Parousis, A. , & Jeschke, M. G. (2018). Pathophysiological response to burn injury in adults. Annals of Surgery, 267, 576–584. 10.1097/SLA.0000000000002097 29408836 PMC8966302

[phy215264-bib-0041] Stanojevic, S. , Graham, B. L. , Cooper, B. G. , Thompson, B. R. , Carter, K. W. , Francis, R. W. , & Hall, G. L. (2017). Official ERS technical standards: Global Lung Function Initiative reference values for the carbon monoxide transfer factor for Caucasians. European Respiratory Journal, 50. 10.1183/13993003.00010-2017 28893868

[phy215264-bib-0042] Stephenson, S. F. , Esrig, B. C. , Polk, H. C. Jr , & Fulton, R. L. (1975). The pathophysiology of smoke inhalation injury. Annals of Surgery, 182, 652–660. 10.1097/00000658-197511000-00020 242281 PMC1344053

[phy215264-bib-0043] Suman, O. E. , Mlcak, R. P. , & Herndon, D. N. (2002). Effect of exercise training on pulmonary function in children with thermal injury. The Journal of Burn Care & Rehabilitation, 23, 288–293. 10.1097/00004630-200207000-00013 12142585

[phy215264-bib-0044] Whitener, D. R. , Whitener, L. M. , Robertson, K. J. , Baxter, C. R. , & Pierce, A. K. (1980). Pulmonary function measurements in patients with thermal injury and smoke inhalation. American Review of Respiratory Disease, 122, 731–739. 6778276 10.1164/arrd.1980.122.5.731

[phy215264-bib-0045] Willis, C. E. , Grisbrook, T. L. , Elliott, C. M. , Wood, F. M. , Wallman, K. E. , & Reid, S. L. (2011). Pulmonary function, exercise capacity and physical activity participation in adults following burn. Burns, 37, 1326–1333. 10.1016/j.burns.2011.03.016 21530086

[phy215264-bib-0046] Wolf, S. E. , Cancio, L. C. , & Pruitt, B. A. (2018). Epidemiological, demographic and outcome characteristics of burns. In D. N. Herndon (Ed.), Total Burn Care. 5th edn. Elsevier. (p. 14–27.e12).

[phy215264-bib-0047] Won, Y. H. , Cho, Y. S. , Joo, S. Y. , & Seo, C. H. (2020). The effect of a pulmonary rehabilitation on lung function and exercise capacity in patients with burn: A prospective randomized single‐blind study. Journal of Clinical Medicine, 9(7), 2250. 10.3390/jcm9072250 PMC7409013 32679866

[phy215264-bib-0048] Won, Y. H. , Cho, Y. S. , Joo, S. Y. , & Seo, C. H. (2021). Respiratory characteristics in patients with major burn injury and smoke inhalation. Journal of Burn Care & Research, 43(1), 70–76. 10.1093/jbcr/irab085 34142710

